# CSDE1 stabilizes AGO2 in embryonic stem cells

**DOI:** 10.3389/fmolb.2025.1745258

**Published:** 2026-01-15

**Authors:** Yuguan Jiang, Mason Waye, Pavan Kumar Kakumani

**Affiliations:** Department of Biochemistry, Memorial University of Newfoundland, St. John’s, NL, Canada

**Keywords:** Ago2, Csde1, embryonic stem cells, pluripotent proteins, ubiquitination

## Abstract

The cold shock domain (CSD)-containing protein, CSDE1, interacts with AGO2 and regulates miRNA function in post-transcriptional gene silencing. While the individual roles of CSDE1 and AGO2 in regulating gene expression underlying stem cell pluripotency and differentiation are well known, the effects of their interaction remain unclear. Here, we demonstrate that CSDE1 stabilizes AGO2 and key pluripotent proteins, NANOG, SOX2, and Oct4, in mouse embryonic stem cells. CSDE1 stabilizes AGO2 and the stem cell markers, preventing their ubiquitination. Further, the N-terminal domain, CSD1, which is necessary for CSDE1 interaction with AGO2, is crucial for maintaining AGO2 levels and the pluripotent proteins, thereby revealing an additional layer of control over AGO2 function and gene expression associated with stem cell fate at the post-translational level.

## 1 Introduction

Argonaute (AGO) proteins are central components of the microRNA (miRNA) pathway, and among the four AGO isoforms identified in humans, only AGO2 has intrinsic endonuclease activity (Nakanishi, 2022). Mature miRNAs are loaded onto AGO2, which forms the miRNA-induced silencing complex to mediate post-transcriptional gene silencing (Gebert and MacRae, 2019). The extent of complementarity between the miRNA and its target mRNA determines the silencing mechanism. Complete complementarity triggers AGO2-mediated cleavage of the target mRNA, whereas partial complementarity typically results in miRISC-mediated translational repression, deadenylation and decapping (Shang et al., 2023). In stem cells, AGO2 has emerged as a regulator of pluripotency (Müller et al., 2020). Notably, knocking out AGO2 in mouse embryonic stem cells (mESCs) increases self-renewal and decreases differentiation (Liu et al., 2024). AGO2 associates with the pro-differentiation miRNA let-7. Elevated AGO2 levels increase let-7 expression and disrupt pluripotency in mESCs (Liu and Chen, 2021). Additionally, AGO2 is highly developmentally regulated among the four isoforms in mESCs, and miR-182 and miR-183 repress AGO2 and regulate stemness in mESCs (Liu and Novak, 2021). Moreover, the AGO2-let-7 interaction downregulates AGO1, a homolog that supports pluripotency, thereby promoting differentiation (Schuh et al., 2025). Despite these critical roles in pluripotency and differentiation, our understanding of how AGO2 is regulated in ESCs is still limited.

Cold Shock Domains (CSDs) are among the most evolutionarily conserved protein domains and are best known for their role in nucleic acid binding (Heinemann and Roske, 2021). Cold Shock Domain-containing protein E1 (CSDE1) is a cytoplasmic RNA-binding protein (RBP) that consists of nine CSDs and plays a critical role in post-transcriptional gene regulation, including translation, mRNA stability, and splicing (Hollmann et al., 2020; Ciocia et al., 2024). CSDE1 was shown to be a component of the miRISC in mESCs and interacts with AGO2 in pluripotent P19 embryonal carcinoma cells, and the miRNA-specific AGO1 in *Drosophila* Embryo Extracts (Kakumani et al., 2020). Interestingly, in mESCs, CSDE1 destabilizes Gata6 mRNA and prevents mESC differentiation into primitive endoderm (Elatmani et al., 2011). In human ESCs (hESCs), CSDE1 levels are elevated, and CSDE1 knockdown decreased expression of key pluripotency markers, such as OCT4, NANOG, and DPPA2, accompanied by increased neural morphology and increased expression of the neurogenesis transcription factor PAX6 (Ju Lee et al., 2017). Additionally, CSDE1 negatively regulates the mRNA levels and translation of radial glial cell markers FABP7 and VIM, thereby preventing neural fate in hESCs (Ju Lee et al., 2017). Together, these findings highlight CSDE1’s regulatory role in stem cell maintenance. And yet, the molecular understanding of its mechanisms controlling gene expression in embryonic stem cells is incomplete.

In the current study, we examined the role of CSDE1 in regulating AGO2 expression in naïve mouse embryonic stem cells. We demonstrated that CSDE1 influences AGO2 stability and pluripotent protein levels by preventing ubiquitination, and that the N-terminal domain of CSDE1, which is required for AGO2 interaction, is crucial for maintaining AGO2 and pluripotent protein levels. Overall, our findings reveal a new layer of post-translational control over AGO2, mediated by CSDE1 in the regulation of stem cell pluripotency.

## 2 Methods

### 2.1 Cell culture

The V6.5 mESC line was established from cells derived from the inner cell mass (ICM) of a 3.5-day-old mouse embryo from a C57BL/6 × 129/sv cross. They were cultured on gelatin (0.2%) coated plates in 2iLIF medium ((0.25X N-2 Supplement (Thermo Fisher), 0.5X B-27 Supplement minus vitamin A (Thermo Fisher), 0.0025% Bovine Albumin Fraction V (Thermo Fisher), 50 U/mL penicillin and 50 μg/mL streptomycin (Sigma-Aldrich), 2 mM L-Glutamine (Thermo Fisher) in 240 mL of Neurobasal Medium (Thermo Fisher) and 244 mL of DMEM/F-12 (Thermo Fisher), supplemented with mouse Leukemia Inhibitory Factor (LIF) (Sigma-Aldrich) (1000 U/mL), MEK/ERK pathway inhibitor, PD 0325901 (1 μM) (Cayman Chemical) and GSK-3 inhibitor CHIR99021 (Sigma-Aldrich) (3 μM)) (Silva et al., 2008). The P19 Embryonal Carcinoma (EC) cell line was isolated from the embryo of a male C3H/He mouse with teratocarcinoma, and it exhibits epithelial-like morphology (ATCC CRL-1825). They were cultured in StableCell MEM (Sigma-Aldrich) supplemented with 7.5% bovine calf serum (Sigma-Aldrich), 2.5% FBS (Wisent), 50 U/mL penicillin, and 50 μg/mL streptomycin (Sigma-Aldrich). The cells were grown in a humidified incubator at 37 °C and 5% CO2.

### 2.2 Transfections

Cells were seeded at 50%–80% confluency in 6-well plates, 24 h prior. siRNA transfection of mESCs and P19 EC cells was performed at 50 nM using Lipofectamine RNAiMAX (Thermo Fisher) and jetPRIME (Polypus) reagents, respectively, according to the manufacturers’ instructions. Plasmid DNA transfection was performed using the jetOPTIMUS reagent (Polyplus) according to the manufacturer’s instructions. In both cases, cells were harvested 48 h post-transfection for downstream experiments.

### 2.3 Western blotting

Cells were lysed using 1x lysis buffer (25 mM Tris-HCl (pH 7.4), 150 mM NaCl, 1% IGEPAL CA-630, 1 mM EDTA, 5% glycerol with cOmplete, Mini EDTA-free protease inhibitors (Sigma-Aldrich)). The lysate was centrifuged at 15,000 g at 4 °C for 15 min, the supernatant was collected, and protein concentration was measured using Bradford Reagent (Bio-Rad). 50 μg of protein samples were run on a 10% SDS polyacrylamide gel, using 1X SDS running buffer at 150V. Once complete, the gel was transferred to a nitrocellulose membrane using the Trans-Blot Turbo transfer system (Bio-Rad) and blocked in 5% skim milk in 1x PBST with gentle agitation at room temperature. Next, the membrane was incubated overnight at 4 °C with gentle agitation in the primary antibody diluted in 1% BSA in 1x PBST, as recommended by the manufacturer. Primary antibodies were acquired as follows: CSDE1 (Abcam, Cat# ab201688), AGO2 (Abcam, Cat# ab186733), β-actin (Abcam, Cat# ab49900), FLAG (Sigma-Aldrich, Cat# F1804), NANOG (Proteintech, Cat# 14295-1-AP), SOX2 (Cell Signaling, Cat# 23064T), OCT4 (Proteintech, Cat# 11263-1-AP), β-tubulin (Proteintech, Cat# 66240-1-IG), Ubi (Santa Cruz, Cat#sc-8017). Following, the membrane was washed with 1x PBST three times and incubated for 45 min in secondary antibody prepared in 1x BSA in PBST at a dilution of 1:2000. Secondary antibodies used were Peroxidase-conjugated AffiniPure Goat Anti-Rabbit IgG (Jackson) and Peroxidase-conjugated AffiniPure Sheep Anti-Mouse IgG (Jackson). The membrane was then washed in 1x PBST and developed with Western Lightning Plus-ECL (PerkinElmer) according to the manufacturer’s instructions. Blots were imaged using a ChemiDoc Touch Gel Imaging System (Bio-Rad) and analyzed in Image Lab 6.1 Software (Bio-Rad).

### 2.4 Real-time PCR

Total RNA extraction was carried out using phenol-based TRIzol reagent (Sigma-Aldrich) following the manufacturer’s instructions. Real-time PCR was performed using the CFX96 Touch Real-Time PCR Detection System (Bio-Rad) and the GoTaq RT-qPCR kit (Promega), with β-actin as the housekeeping gene. Gene-specific primers for AGO2 and β-actin were designed and acquired using IDT’s PrimerQuest tool. Data were collected from three independent cell culture transfection experiments. Relative gene expression for each experiment was quantified, and statistical significance was determined using an independent-samples t-test.

### 2.5 Co-immunoprecipitation

Dynabeads Protein G (Thermo Fisher) (20 μL for 2 mg of total protein extract and 10 μg of antibody) were used for co-immunoprecipitation with specific antibodies as previously described (Ramakrishna et al., 2025). Briefly, cell lysates were pre-cleared by incubation with Dynabeads at 4 °C, then transferred to Eppendorf tubes containing the antibody-bead complex and incubated overnight at 4 °C. Following incubation, the beads were washed three times with 1X lysis buffer, and samples were then extracted for Western blotting by adding 1X SDS loading buffer or for RNA extraction using TRIzol reagent (Sigma-Aldrich).

## 3 Results

### 3.1 CSDE1 controls AGO2 and pluripotent protein expression in naïve mESCs

**FIGURE 1:**
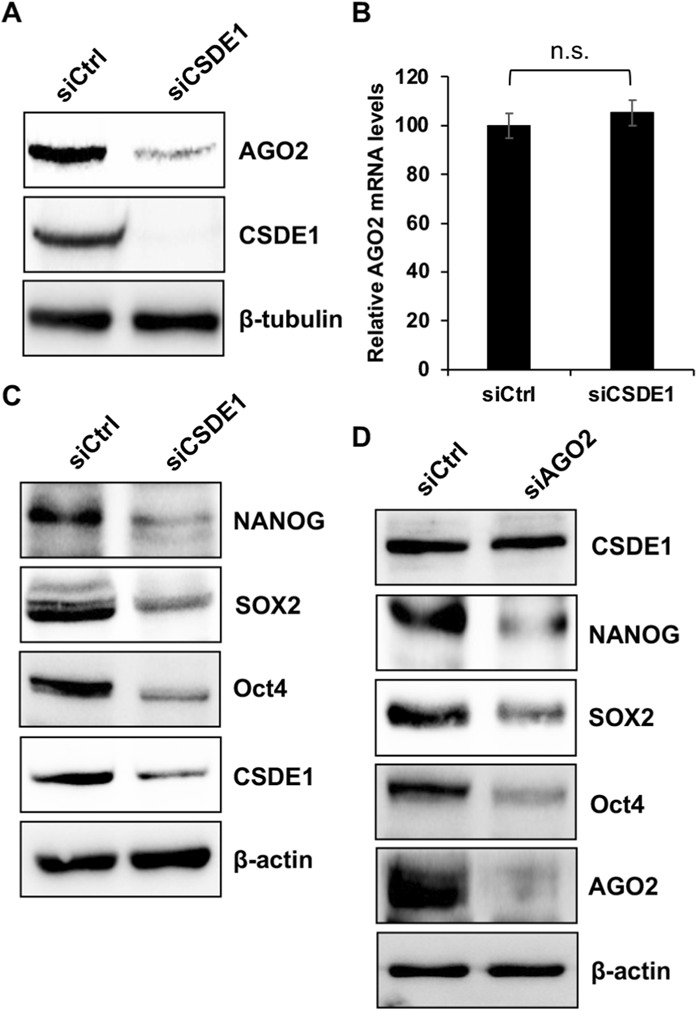
Loss of CSDE1 downregulates AGO2 and pluripotency markers in naïve mESCs. **(A–C)** Western blot showing the expression of AGO2 **(A)** and pluripotent protein markers **(C)** in V6.5 cells under Control and CSDE1 knockdown conditions. **(B)** Relative AGO2 mRNA levels in total RNA from V6.5 cells under Control and CSDE1 knockdown conditions. **(D)** Western blot analysis of CSDE1 and pluripotent protein expression in V6.5 cells under Control and AGO2 knockdown conditions. In Western blotting, β-actin and β-tubulin were used as loading controls. For relative quantification of gene expression by real-time PCR, β-actin was used as the housekeeping gene. Data are presented as mean ± SD (n.s., not significant, p > 0.05, n = 3, two-tailed t-test).

CSDE1 interacts with the AGO2-miRISC in mESCs (Kakumani et al., 2020). To further understand the relationship between CSDE1 and AGO2 in stem cells, we investigated the effects of CSDE1 knockdown on AGO2 expression in V6.5 cells cultured in a basal, serum-free medium supplemented with 2iLIF. The AGO2 protein levels were downregulated under CSDE1 knockdown conditions compared to the control, with no significant change in its mRNA levels (Figures 1A,B). However, the effects on AGO2 protein expression observed with CSDE1 depletion were lost in pluripotent P19 cells derived from teratocarcinomas (Supplementary Figures S1A–B). Also, we observed decreased expression of NANOG, SOX2, and Oct4 in mESCs under CSDE1-depleted conditions compared to the control (Figure 1C). However, this pattern is absent in P19EC cells (Supplementary Figure S1C), indicating that CSDE1-mediated regulation of AGO2 and the pluripotent proteins is specific to naïve mESCs. Further, as AGO2 has been shown to regulate pluripotency and stem cell differentiation in serum-containing media (Liu and Chen, 2021; Liu and Novak, 2021; Liu et al., 2024), we investigated whether AGO2 regulates pluripotency at the molecular level in V6.5 cells. The expression levels of NANOG, SOX2, and Oct4 were decreased in cells depleted of AGO2 compared with control conditions, whereas CSDE1 protein levels remained unchanged (Figure 1D). However, these effects were lost in P19EC cells (Supplementary Figure S1D). Collectively, our results show that the regulation of pluripotent gene expression is unidirectional and controlled by CSDE1, suggesting that CSDE1 and AGO2 are crucial for maintaining the gene expression underlying pluripotency in mESCs but are unlikely to be required in stem cells derived from carcinomas.

### 3.2 CSDE1 regulation of AGO2 and pluripotent factors is associated with protein degradation pathways

**FIGURE 2:**
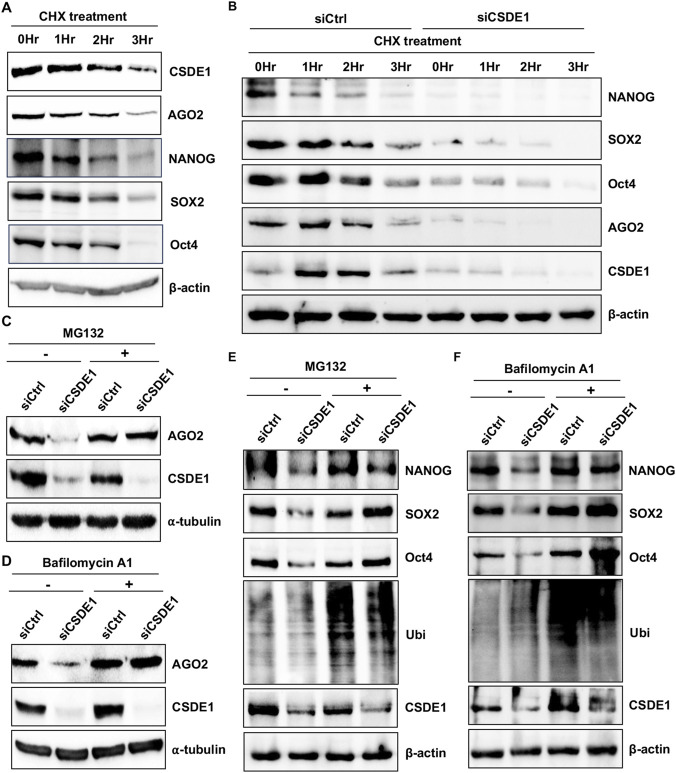
CSDE1 negatively regulates AGO2 and pluripotent proteins via lysosomal and proteasomal degradation. **(A,B)** Western blots for the indicated proteins from V6.5 mESCs treated with CHX for a period of 3 h, and cells collected at 0 h, 1 h, 2 h, and 3 h post-treatment **(A)**, and under Control and CSDE1 knockdown conditions **(B)**. **(C–E)** Western blot analysis of AGO2 **(C)** and the pluripotency markers, along with ubiquitin (Ubi) **(E)**, in V6.5 mESCs treated with the proteasomal inhibitor, MG132 (+), or DMSO (−), under Control and CSDE1 knockdown conditions. **(D–F)** Western blot analysis of AGO2 **(D)** and the pluripotency markers along with Ubi **(F)** in V6.5 mESCs treated with the lysosomal inhibitor, Bafilomycin (+), or DMSO (−), under Control and CSDE1 knockdown conditions. α-tubulin and β-actin were used as loading controls.

To study the degradation of AGO2 and the pluripotent proteins in the absence of CSDE1, we employed cycloheximide (CHX) chase assays. mESCs treated with CHX over a period of 3 hours showed a time-dependent decrease in the levels of CSDE1, AGO2 and the stem cell proteins NANOG, SOX2 and Oct4 (Figure 2A). However, there was no discernible difference in either CSDE1 or AGO2 or the pluripotent proteins under the same experimental conditions in P19 EC cells (Supplementary Figure S2A). We also repeated the CHX chase assay using mESCs knocked down for CSDE1 expression. We observed that CSDE1 loss downregulated AGO2 and pluripotent protein levels more rapidly than in control conditions (Figure 2B), indicating that CSDE1 mediates the stability of these proteins. Following the correlation between target protein expression, we performed CSDE1 knockdowns in mESCs treated with proteasomal or lysosomal inhibitors, MG132 and Bafilomycin A1, respectively, and measured the expression of AGO2 and pluripotent proteins. AGO2 protein levels were downregulated in the CSDE1 knockdown but were restored upon treatment with MG132 or Bafilomycin A1, compared to mock treatment with DMSO (Figures 2C,D). We also observed a similar pattern for the pluripotent markers NANOG, SOX2, and Oct4 under the same experimental conditions (Figures 2E,F), with the band pattern (seen in the case of blots probed with Ubi antibody) reflecting the accumulation of ubiquitinated proteins under the treatment conditions, confirming the blockade of the respective pathways. In contrast, there was no difference in AGO2 levels in either the CSDE1 knockdown condition, with or without inhibitor treatment, in P19EC cells (Supplementary Figure S2B,C). Together, these findings demonstrate that the ubiquitin system is a key degradation pathway for AGO2 and pluripotency regulators governed by CSDE1 in mESCs.

### 3.3 CSDE1 maintains AGO2 and pluripotent protein levels through ubiquitination

**FIGURE 3:**
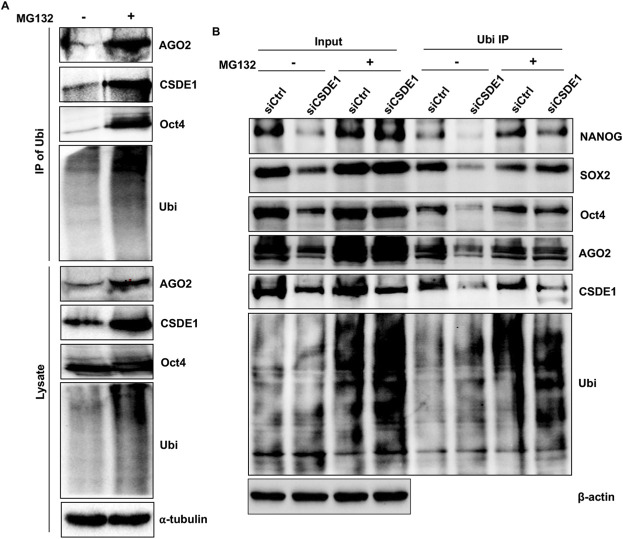
CSDE1 regulates AGO2 and pluripotent proteins in mESCs via ubiquitination. **(A)** Immunoprecipitation of Ubi using anti-Ubi antibody was performed with lysate from V6.5 mESCs treated with MG132 (+) or DMSO (−). Western blotting was performed to probe for the indicated proteins. **(B)** Western blot analysis of indicated proteins in the total lysate (input) and the Ubi IPs from Control and CSDE1 knockdown conditions treated with MG132 (+) or DMSO (−). α-tubulin and β-actin were used as loading controls.

**FIGURE 4:**
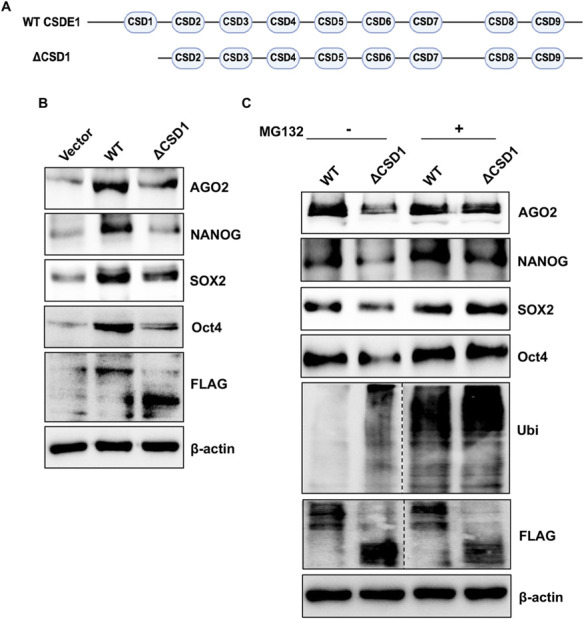
The N-terminal domain, CSD1, is necessary to maintain AGO2 and pluripotent protein levels. **(A)** Schematics of the CSDs in WT CSDE1, and the deletion mutant, ΔCSD1. **(B)** Western blots showing the expression of indicated proteins in V6.5 mESCs transiently expressing FLAG-tagged WT CSDE1, the deletion mutant ΔCSD1, or the vector control. **(C)** Western blot analysis of indicated proteins in the total lysate (input) and samples from the IP of Ubi using V6.5 mESC lysate transiently expressing FLAG-tagged WT CSDE1, or the deletion mutant, treated with MG132 (+) or DMSO (−). β-actin was used as a loading control.

Post-translational regulation by ubiquitination is crucial for mESC self-renewal and for controlling differentiation (Strikoudis et al., 2014). Since ubiquitination is a common mechanism for targeting substrates to the proteasomal and lysosomal degradation pathways in mammalian cells (Clague and Urbé, 2010), we investigated whether CSDE1’s regulation of AGO2 stability and pluripotent proteins is mediated by ubiquitination. In mESCs treated with the proteasomal inhibitor that blocks the degradation of ubiquitylated proteins, we observed an increased expression of CSDE1 and AGO2 compared to mock-treated cells (Figure 3A). Additionally, as expected, Oct4 levels were elevated, accompanied by a noticeable increase in ubiquitylated proteins in the total lysate (Figure 3A). Furthermore, examining the fraction of ubiquitylated proteins through immunoprecipitation of Ubi from the treated lysate confirmed that both CSDE1 and AGO2 are highly ubiquitylated, similar to Oct4 in mESCs (Figure 3A). To validate further that the ubiquitination of AGO2 and Oct4 is indeed related to CSDE1, we performed CSDE1 knockdowns in mESCs, treated with MG132, and examined their expression pattern. As shown in Figure 3B, ubiquitination of AGO2, NANOG, SOX2 and Oct4 is higher under CSDE1 knockdown conditions than in the control, indicating that CSDE1 regulates AGO2 and the pluripotent proteins via post-translational ubiquitination. Next, to assess the impact of CSDE1 and AGO2 interaction on AGO2 ubiquitination and stability, we transiently expressed the WT CSDE1 and the deletion mutant ΔCSDE1 in V6.5 cells. We measured AGO2 and pluripotent protein expression. The levels of AGO2, NANOG, SOX2, and Oct4 were increased in WT CSDE1. In contrast, cells expressing ΔCSD1 showed no change, similar to the vector control conditions (Figures 4A,B). Also, we observed no significant difference in their mRNA levels in WT CSDE1-expressing cells compared to the vector control (Supplementary Figure S3). Further, mESCs expressing WT CSDE1 or ΔCSD1 and treated with MG132 showed that AGO2 and pluripotent protein levels were restored in the ΔCSD1 line, similar to WT CSDE1 (Figure 4C). We also observed an accumulation of ubiquitylated proteins in ΔCSD1-expressing cells compared to WT CSDE1. Together, our results revealed that CSDE1 modulates AGO2 protein levels via ubiquitination and that the N-terminal CSD, CSD1, which is necessary for interaction with AGO2, is crucial for controlling AGO2 and pluripotent protein ubiquitination in mESCs.

## 4 Discussion

Within the miRISC, AGO2 serves as the key RBP that binds miRNAs to target mRNAs for silencing gene expression (Gebert and MacRae, 2019), and CSDE1 interacts with AGO2-miRISC in mESCs (Kakumani et al., 2020). In the current study, we show that CSDE1 stabilizes AGO2 protein (Figures 1A,B) and, consistent with previous studies (Ju Lee et al., 2017), it also influences the expression of pluripotency markers, namely NANOG, SOX2 and Oct4 in mESCs (Figure 1C). However, this relationship is absent in tumour-derived pluripotent P19 EC cells (Supplementary Figure S1), thereby identifying CSDE1 as an upstream regulator of AGO2 and pluripotent proteins in naïve mESCs but not in embryonal carcinomas with stem cell features, likely due to their origin from tumors with malignant properties.

Given that both CSDE1 and AGO2 have been previously implicated in controlling pluripotency in ESCs (Ju Lee et al., 2017; Liu et al., 2024), it is not surprising that CHX treatment led to a marked reduction of CSDE1 within 3 hours, which correlated with the expression of AGO2 and the pluripotent proteins (Figure 2A), suggesting a coordinated regulation of their stability linked to stem cell homeostasis. We also observed a rapid reduction in AGO2 and pluripotent protein levels in CSDE1-depleted mESCs under CHX treatment (Figure 2B), demonstrating that CSDE1 stabilizes AGO2 and pluripotent proteins. Interestingly, in animals, AGO protein degradation occurs through the proteasome and lysosomal pathways (Bressendorff et al., 2025). Accordingly, inhibition of these pathways under CSDE1-depleted conditions restored AGO2 levels in mESCs (Figures 2C,D). Further, findings from the study by Bressendorff et al. identified that the conserved N-coil of AGO2 acts as a structural switch that enables rapid proteolysis (Bressendorff et al., 2025) and, as such, can provide a possible structural basis for CSDE1-mediated regulation of AGO2. Similarly, inhibition of the proteasome (Figure 2E) or the lysosomal (Figure 2F) pathways rescued NANOG, SOX2, and Oct4 expression, suggesting that these factors are degraded via similar mechanisms under CSDE1-depleted conditions, likely involving ubiquitination. While full half-life curve quantification would further strengthen this point, the present data demonstrate directionally consistent effects supporting decreased AGO2 stability via ubiquitination following CSDE1 loss. It presents a coherent model in which CSDE1 loss promotes AGO2- and pluripotent protein-mediated ubiquitination and turnover, and lays the foundation for future detailed biochemical characterization.

Ubiquitin-mediated protein turnover is a fundamental mechanism that maintains stem cell identity by precisely controlling the levels of core pluripotency regulators (Strikoudis et al., 2014). Our observation that CSDE1 and AGO2 are also ubiquitinated in mESCs (Figure 3A) and that loss of CSDE1 enhances ubiquitination of AGO2 and pluripotency factors (Figure 3B) suggests that CSDE1 is an integral component of this post-translational quality-control system. Rather than functioning solely as an RBP, CSDE1 may act as a molecular safeguard that coordinates protein stability and RNA regulatory activity through its interaction with AGO2. This relationship points to a broader model in which the CSDE1–AGO2 axis links ubiquitin-dependent protein quality control with the maintenance of stem cell pluripotency, ensuring that gene expression programs remain tightly regulated during early developmental states.

Consistent with AGO2’s role in sustaining the stem cell transcriptome, we observed that AGO2 knockdown led to reduced expression of NANOG, SOX2, and OCT4 (Figure 1D). Interestingly, this result contrasts with previous studies reporting weaker or even inverse effects of AGO2 loss on pluripotency marker expression (Müller et al., 2020; Liu and Novak, 2021; Liu et al., 2024). A likely explanation for this discrepancy lies in differences in the stem cell lines and culture conditions used. Our experiments employed V6.5 mESCs maintained under serum-free 2iLIF conditions, which support a more naïve pluripotent state with reduced signalling heterogeneity (Silva et al., 2008). In contrast, earlier studies typically used E14 cells grown in serum-containing medium, representing a more primed and heterogeneous state (Liu and Novak, 2021; Liu et al., 2024). These distinct culture environments impose different signalling and metabolic landscapes that can reshape post-transcriptional regulation and protein turnover, thereby influencing how AGO2 controls stem cell gene expression. Thus, the regulatory function of AGO2 in maintaining pluripotency is likely context-dependent, modulated by the pluripotent state and its associated molecular circuitry. Notably, although AGO2 knockdown destabilizes pluripotency factors, CSDE1 levels remain unchanged (Figure 1D), raising the question of whether CSDE1 regulates these proteins directly or via AGO2. Our data point to a requirement for CSDE1–AGO2 interaction in maintaining pluripotency protein stability, as disruption of this interaction through deletion of the CSD1 domain similarly destabilizes NANOG, SOX2, and OCT4 (Figures 4B,C).

In line with previous studies on CSDE1’s role in promoting stemness, overexpression of wild-type CSDE1 increased the expression of NANOG, SOX2 and Oct4, with no significant change in their mRNA levels (Figure 4A; Supplementary Figure S3) (Ju Lee et al., 2017). Conversely, the deletion mutant ΔCSD1 behaved similarly to the vector control. Interestingly, our rescue experiments with AGO2 in CSDE1 knockdown mESCs did not show a robust or consistent restoration of NANOG, SOX2, or Oct4 protein levels (Supplementary Figure S4), indicating that simply increasing AGO2 expression is insufficient to counteract the effects of CSDE1 loss and is consistent with a requirement for functional interaction between CSDE1 and AGO2. This supports the idea that CSDE1 facilitates the recruitment of AGO2 to mRNAs encoding components of the ubiquitin–proteasome system, such as HECW2 (Hynes et al., 2026), thereby indirectly influencing proteasomal degradation of pluripotency regulators.

Given AGO2’s central role in miRNA-guided gene silencing (Gebert and MacRae, 2019), the ubiquitination of AGO2 and its regulation by CSDE1 (Figures 3, 4) likely represent an intersection between post-transcriptional and post-translational control in pluripotent stem cells. By stabilizing AGO2, CSDE1 is well positioned to influence the efficacy and durability of miRNA-mediated gene silencing programs that are essential for maintaining pluripotency. Disruption of this axis may therefore compromise miRNA-dependent repression of differentiation-associated transcripts, linking AGO2 stability directly to stem cell identity. CSDE1 has been implicated in the regulation of mRNA stability and translation of ubiquitin ligases, such as HECW2 (Hynes et al., 2026), suggesting that it may influence protein homeostasis by modulating the ubiquitination machinery. Since CSDE1 interaction with AGO2 via CSD1 is key to maintaining AGO2 stability (Figure 4) (Kakumani et al., 2020), future work using AGO2 miRNA-binding mutants in mESCs will be particularly informative in determining whether the observed post-translational effects depend on miRNA binding of AGO2 or not, as reported by Liu Q et al., 2024, in the case of AGO1 through interactions with heat shock protein Hsp70 and Hsp90 through protein folding. If the loss of the CSDE1–AGO2 interaction derepresses or helps stabilize a ubiquitin ligase targeting AGO2, OCT4, SOX2, or NANOG, it would uncover a feedback mechanism in which CSDE1–AGO2 cooperatively stabilize the stem cell proteome by coupling miRNA function to protein quality control. This model highlights an underexplored layer of gene regulation in which RBPs such as CSDE1 integrate miRNA activity with ubiquitin-dependent proteostasis to sustain pluripotency.

## Data Availability

The original contributions presented in the study are included in the article/Supplementary Material. Further inquiries can be directed to the corresponding author.
