# Supplementary material for: CSDE1 stabilizes AGO2 in embryonic stem cells
Source: Front Mol Biosci. 2026 Jan 15;12:1745258. doi: 10.3389/fmolb.2025.1745258 (PMC12851991; doi:10.3389/fmolb.2025.1745258)
Supplement: Supplementary file 1 [file Supplementaryfile1.docx]

**Supplementary material**

**1. Supplementary Figures**

**
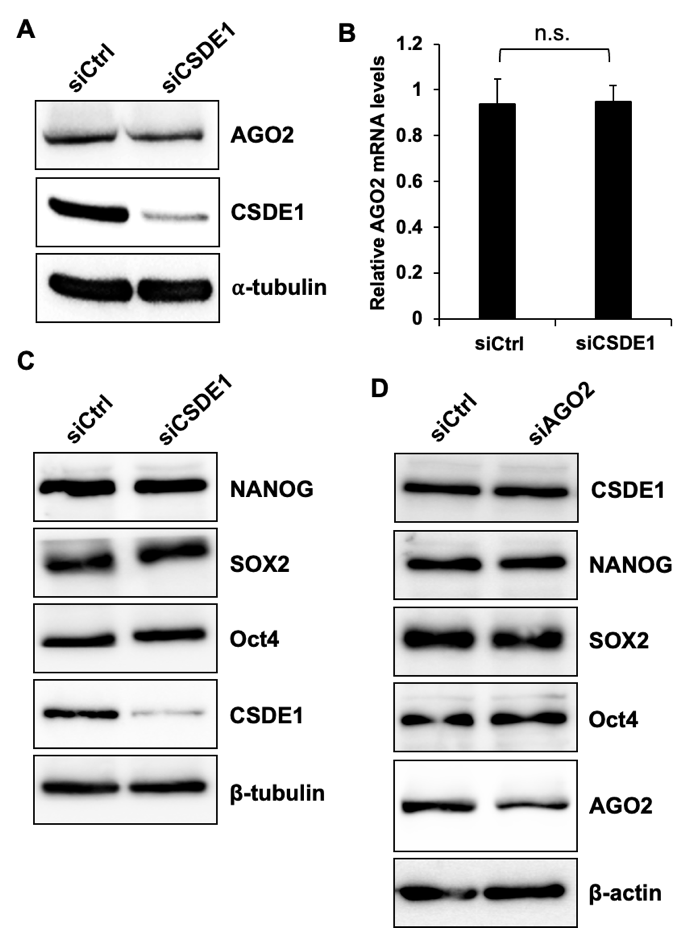
**

**Supplementary Figure 1: Knockdown of CSDE1 or AGO2 does not alter the expression of pluripotent proteins in P19 EC cells.** (A, C) Western blot showing the expression of AGO2 (A) and pluripotent proteins (C) under Control and CSDE1 knockdown conditions. (B) Relative AGO2 mRNA levels in total RNA under Control and CSDE1 knockdown conditions. (D) Western blot analysis of CSDE1 and pluripotent protein expression under Control and AGO2 knockdown conditions. In western blotting, ⍺-tubulin and β-actin were used as loading controls. For relative quantification of gene expression by real-time PCR, β-actin was used as the housekeeping gene. Data are presented as mean ± SD (n.s., not significant, p> 0.05, n = 3, two-tailed t-test).

**
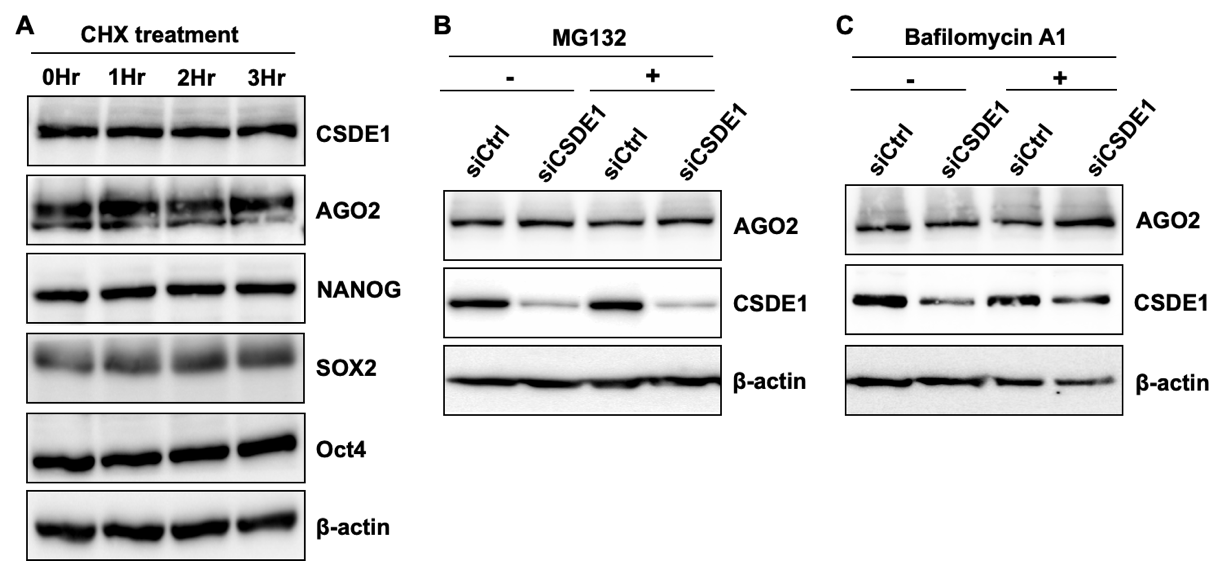
**

**Supplementary Figure 2:** (A) Western blot showing the expression of AGO2 and pluripotent proteins in P19 EC cells treated with CHX for a period of 3 hours, and cells collected at 0h, 1h, 2h, and 3h post-treatment. (B, C) The expression of AGO2 was measured by western blotting using lysates from P19 EC cells treated with MG132 (B) or Bafilomycin (C) under Control and CSDE1 knockdown conditions. β-actin was used as a loading control.

**
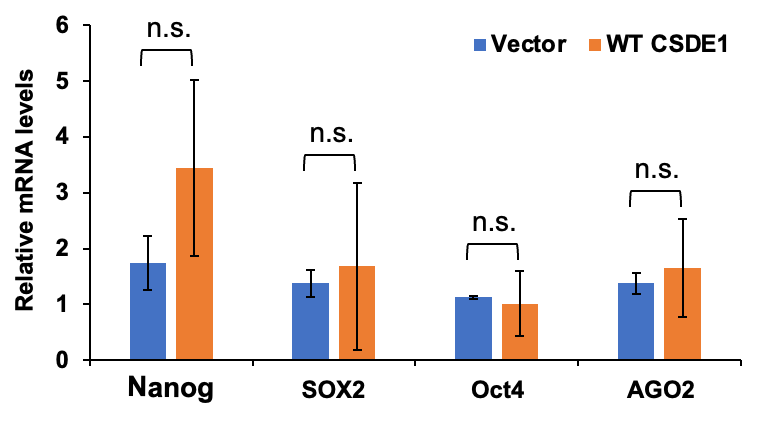
**

**Supplementary Figure 3:** Relative mRNA levels of AGO2 and pluripotency factors in total RNA from V6.5 mESCs transiently expressing FLAG-tagged WT CSDE1 or a vector control. For the relative quantification by real-time PCR, β-actin was used as the housekeeping gene. Data are presented as mean ± SD (n.s., not significant, p> 0.05, n = 3, two-tailed t-test).

**
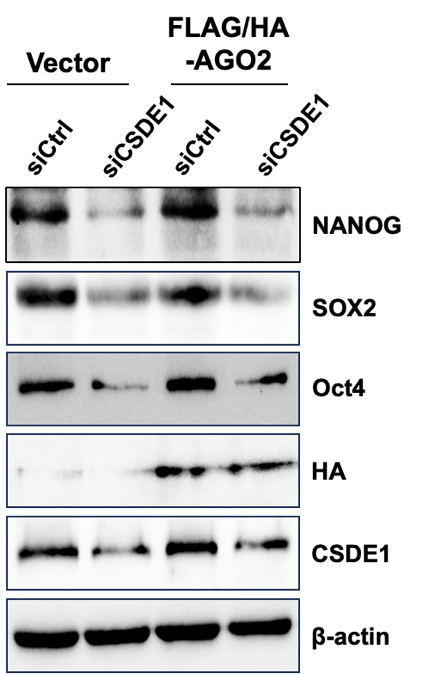
**

**Supplementary Figure 4:** Western blot analysis of indicated proteins in V6.5 mESCs knocked down for CSDE1 and transiently expressing FLAG/HA-tagged AGO2 or not. β-actin was used as a loading control.
